# Thermoplastic Electromagnetic Shielding Materials from the Integral Recycling of Waste from Electronic Equipment

**DOI:** 10.3390/polym15193859

**Published:** 2023-09-22

**Authors:** Mihaela Aradoaei, Romeo C. Ciobanu, Cristina Schreiner, Andrei George Ursan, Elena Gabriela Hitruc, Magdalena Aflori

**Affiliations:** 1 Department of Electrical Measurements and Materials, Gheorghe Asachi Technical University, 700050 Iasi, Romania; mihaela.aradoaei@academic.tuiasi.ro (M.A.); cristina-mihaela.schreiner@academic.tuiasi.ro (C.S.); andrei_urs@yahoo.com (A.G.U.); 2 Petru Poni Institute of Macromolecular Chemistry, 41A Aleea Gr. Ghica Voda, 700487 Iasi, Romania; gabihit@icmpp.ro

**Keywords:** integral recycling of WEEE, thermoplastic composites, electromagnetic interference, electromagnetic compatibility

## Abstract

The European Green Deal’s goals are anticipated to be fulfilled in large part thanks to the New Circular Economy Action Plan. It is believed that recycling materials will have a significant positive impact on the environment, particularly in terms of reducing greenhouse gas emissions and the impacts this will have on preventing climate change. Due to the complexity of the issue and its significant practical ramifications, the activity of Waste Electrical and Electronic Equipment (WEEE) collection networks is a subject of interest for researchers and managers, in accordance with the principles that recent laws have addressed in a large number of industrialized countries. The goal of this paper is to characterize and obtain composite materials using an injection process with a matrix of LDPE, PP, and HDPE, with up to a 10% addition of nonmetallic powders from PCBs and electronic parts from an integrated process of WEEE recycling. The composites present relevant thermal, electrical, and mechanical properties. Such composite materials, due to their relevant dielectric properties, may be further tested for applications in electromagnetic shielding at frequencies above 1 kHz, or for electromagnetic interference/electromagnetic compatibility (EMI/EMC and ESD) applications at lower frequencies due to their superior dielectric loss factor values, associated with relevant behaviors around exploitation temperatures, mainly for the electric, electronic, or automotive industries.

## 1. Introduction

In several domains of application nowadays, plastic materials are employed more often in daily life. After use, these plastic materials are dumped outside. As the majority of plastics are nonbiodegradable by nature, these are known as plastic wastes and are bad for the environment. Therefore, in order to safeguard our environment, the effective treatment of these waste products is required. There are numerous methods for managing these plastic wastes, including recycling, land filling, and degradation (including thermo-, photo-, bio-, and chemo-degradation). Currently, a sizable amount of the plastic trash that is produced is either burnt or dumped in landfills, resulting in both resource loss and environmental pollutants, most notably CO_2_ from burning. Global CO_2_ emissions from the life cycle of plastics are estimated at 400 million tonnes per year (2012) [1,2]. If the current trends continue, by 2050, it may account for 20% of the world’s oil consumption and 15% of the carbon emissions produced annually around the world. The plastics strategy is a milestone of the New Circular Economy since it addresses these issues. The core of this strategy is the packaging, reuse, and recycling of plastic garbage [3,4].

Although the materials used in electrical and electronic applications have significant value and a wide range of uses, they suffer from severe electromagnetic interference (EMI) [5,6,7,8]. Broadcasting, the medical area, research, the defense industry, communication, and other businesses can all benefit greatly from the use of these materials. With the use of efficient EMI shielding materials, the EMI can be controlled. Recent works described different methods for creating EMI shielding from industrial waste, agricultural waste, and other wastes. The procedure for gathering end-of-life products and moving them to designated treatment facilities is acknowledged as a crucial task because its effective execution ensures the reduction of both the consumption of virgin materials and the dissemination of hazardous substances in soil, water, and air [9,10,11]. The requirements must be met in terms of electrical equipment and device emissions as well as their resistance to disruptions. When electrical equipment and devices are connected or placed near to each other, they have an impact on one another. Electromagnetic compatibility (EMC) is the process of ensuring that electrical devices or equipment are both immune to and do not cause disturbances that would impact other devices or equipment [12,13,14]. Due to the wide range of hazardous materials (lead, chlorofluorocarbons, fluorescent dust, etc.) and the difficulties involved in the recovery process, where frequently specialized technologies and controlled-atmosphere storage and treatment are required, waste electrical and electronic equipment (WEEE) recycling is a subject of reticence for high-value applications [15,16,17,18].

Previous research on the purposes of EMI/EMC and antistatic shielding applications has solely involved selected virgin polymer matrices and tailored conductive or ferritic powders for manufacturing relevant composites for electric, electronic, or automotive use.

Such examples may employ virgin PE/carbon nanotube (CNT) composites as EMI shielding materials with three distinct CNT kinds and various network architectures [19], with segregated network-structured composites with an efficiency of 46 dB with only 5 wt% CNT loading.

In all, studies related to the use of recycled PE as a matrix for antistatic and EMI shielding composites are extremely limited, and studies on recycled PP as a matrix are missing due to the structure and quality of polymeric waste streams that need to be improved using a variety of techniques, including restabilization, rebuilding, compatibilization, and the inclusion of elastomers and specialized fillers [20,21,22,23,24,25]. Because thermo-mechanical degradation via multiple processing affects polyolefins (HDPE and PP) more than thermo-oxidative aging does, special attention should be paid to controlling the processing conditions during mechanical recycling [26,27,28,29,30,31]. On the other hand, if recycled polyolefins from packaging are used as the matrices for composites for electromagnetic shielding applications, expensive conductive or ferritic powders are added to achieve the imposed dielectric properties; a clear drawback, because the economy of using recycled matters becomes insignificant in the cost of the final product [2,32,33,34,35,36,37].

This article describes a fully innovative process of the integral recycling of waste from electronic equipment towards manufacturing thermoplastic electromagnetic shielding composites. The advantage lies in a more uniform source of recycled polyolefins (LDPE, HDPE, and PP), which do not need extra processing to increase their thermal, electrical, and mechanical properties. Another advantage lies, on the other side, in the manufacturing of cheap powders obtained from the non-metallic thermoset components of WEEE (electronic components) to substitute expensive conductive or ferritic powders, due to them having homologue dielectric features.

## 2. Materials and Methods

### 2.1. Integral Recycling of WEEE

Plastic wastes should be given fresh thought as valuable resources for product manufacturing, on par with virgin oil-based plastics and biologically derived polymers [38,39,40]. The end-of-life application sector, from which the plastic waste streams originated, dictates the technological techniques to be used for the successful recycling of their plastic wastes. According to their sources, the following categories have been suggested for classifying plastic waste streams: packaging, agriculture, municipal solid waste, construction and demolition, end-of-life vehicles, and waste electrical and electronic equipment (WEEE).

**Figure 1:**
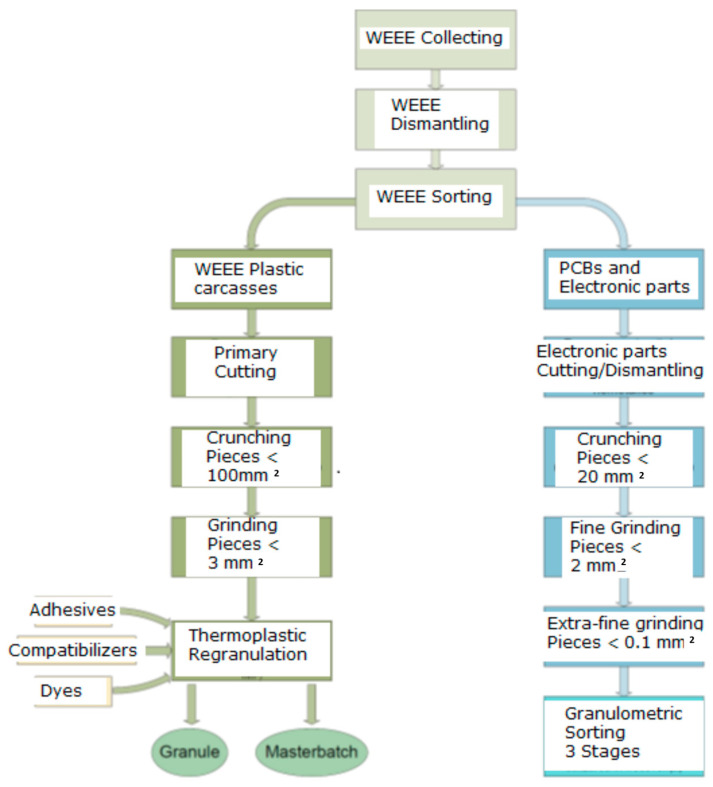
Technological process for the integral recycling of WEEE.

Under the concept of a circular economy, it is a must to develop technologies for the integral use of WEEE towards innovative applications due to the larger and larger quantities of WEEE, which, according to novel electronic technologies, cannot be recycled via classical ways, which mainly recover the metallic components and only partially recover the thermoplastic carcass components, practically ignoring the main parts of the thermoset components after the partial recovery of precious metals. Our paper suggests an integrated recycling of the thermoplastic and non-metallic components of WEEE, according to the scheme in Figure 1, after all classical recycling processes are accomplished.

### 2.2. Powder Obtaining from Thermoset Components of WEEE (Electronic Components)

Such components as the rest of the printed circuit boards, integrated circuits, diodes, capacitors, resistors, etc., exclude metallic, glass, and ferritic items, which are separately selected in a preliminary process, due to them having separate recycling stages in ways that have been demonstrated to be profitable.

Grinding was carried out using a SPEX type mill, 8000 M series (SPEX Europe, Rickmansworth, UK). The grinding time was 4 h, and the rotation speed was 875 cycles/min. Grinding is done in metal or ceramic mini containers made of steel, tungsten carbide, alumina, zirconium, or silicon nitride. Dry grinding is the simplest method to use. For samples that tend to form lumps during mixing or grinding, a particle accelerator can be used. Water, alcohol, or other liquids can also be used. It is recommended to use a fluorocarbon fluid that does not chemically affect the sample and evaporates quickly after use. Water can be easily removed via heating in plastic boxes in an oven at a low temperature.

**Figure 2:**
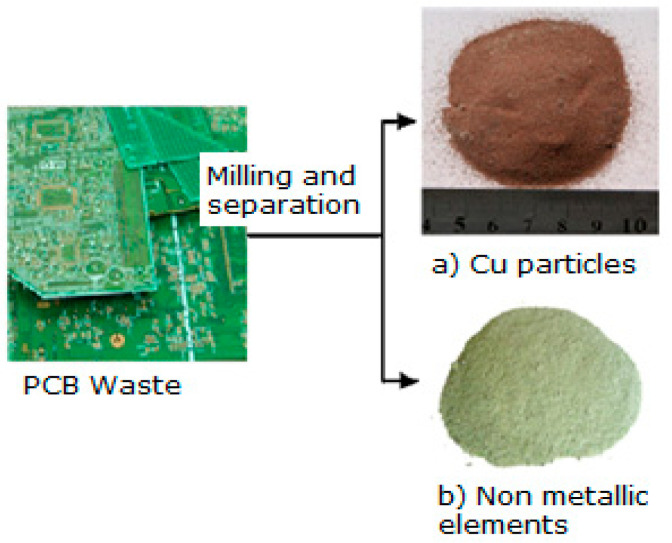
Non-metallic powder aspect after final metallic separation.

The powder aspect after the final metallic separation is shown in Figure 2.

The freely poured density of the powder (ρ) was calculated according to the standards SR ISO 3923-1 [41] and SR ISO 3923-2 [42] (Table 1).

The equipment used to perform X-ray diffraction analyses on powders was an X-ray diffractometer type D8 ADVANCE.

**Figure 3:**
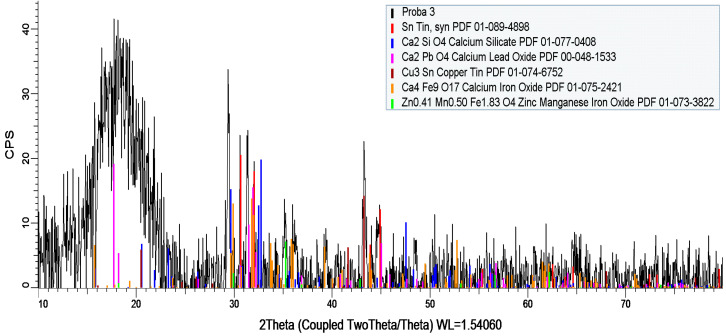
X-ray diffraction spectra of the powder.

Via X-ray diffraction analysis, it was found that the powder consisted of a mixture of metal oxides (CaO, Fe_2_O_3_, CuO, SiO_2_, SnO_2_, PbO, BaO, Br, Cr2O_3_, ZnO, MnO, NiO, ZrO_2_, SrO, Ag) and metals (Pb, Sn, Au, Ag, Si, Ge) in significant concentrations, as given in Table 2 and Figure 3. The total concentration of the metal-derived compounds was approximately 88.32%. Of the rest, up to 100% is expected to be of a polymeric component (thermoset covers of integrated circuits and parts of printed circuit boards). In all, according to the components, the powder presented features related to nanoconductivity, an aspect that is very important when using such powders as additives within recycled polyolefins towards the manufacturing of thermoplastic electromagnetic shielding materials. This aspect will be demonstrated when analyzing the dielectric properties of the composites.

Scanning electron microscopy (SEM) analysis was performed with the help of the FESEM-FIB workstation, Auriga model, produced by Carl Zeiss, Göttingen, Germany, through the secondary electron/ion detector (SESI) in the sample room for the topographic study of the surface. The determination of the chemical composition was carried out with the help of the EDS (energy-dispersive spectrum for characteristic X-ray) probe produced by Oxford Instruments (Oxford, UK), model Inca PET X3. The composition was cooled with liquid nitrogen, and integrated on the FESEM-FIB Auriga workstation. The analyses were carried out in accordance with the Auriga Smart SEM V05.04 Workstation Manual [43].

**Figure 4:**
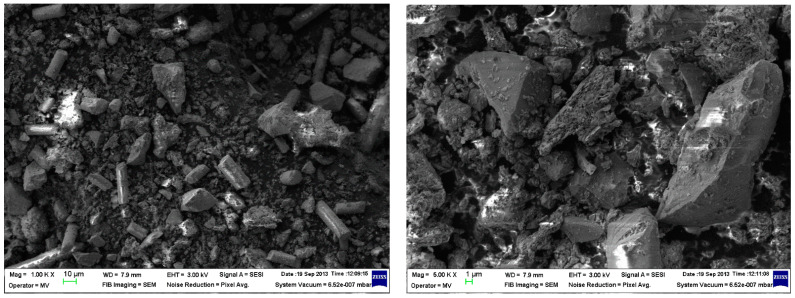
The powder SEM images at 1000× and 5000× magnitude.

The powder dimension depends on the milling process of the WEEE; in our case, we anticipated a quasi-uniform dispersion with dimensions of about 25 μm on average (Figure 4).

**Figure 5:**
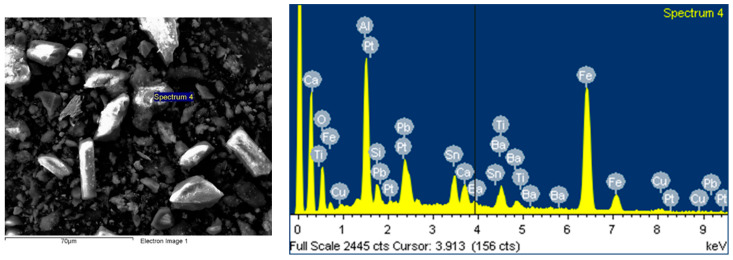

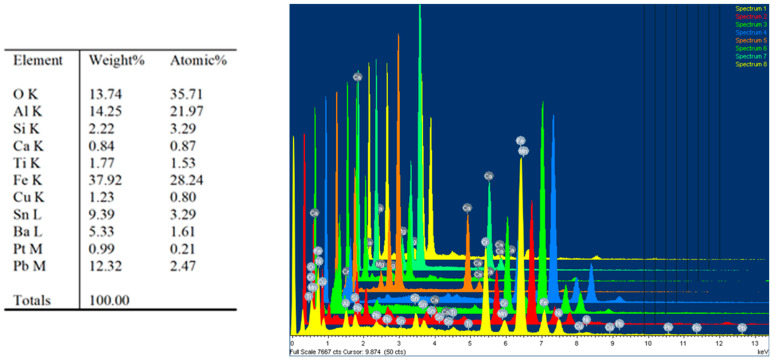
Example of comparative sectorial analysis of powder components.

According to Figure 5, a comparative sectorial analysis of the powder components was made via energy dispersive X-ray spectroscopy upon the 8 random areas of analysis, in order to have a complete and an average value of the components, which are metals and metallic oxides. The results are presented in the table of Figure 5, and are in line with the results from Table 2.

### 2.3. Composites Obtained from Recycled Polyethylene and Powder from WEEE

The physical–chemical and mechanical properties of the LDPE/HDPE/PP matrices used are similar with the ones obtained from the same WEEE sources (WEEE carcasses) [44,45,46,47,48].

**Figure 6:**
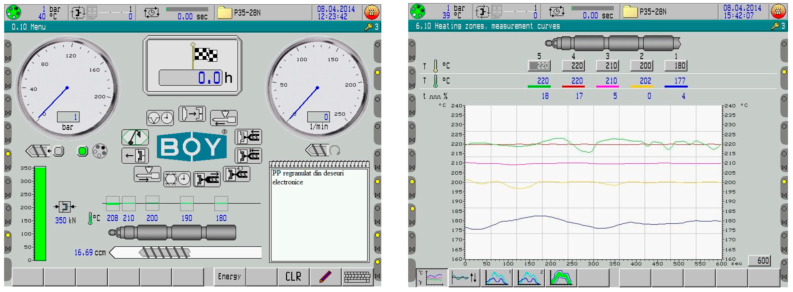
The interface and thermal diagrams of the injection machine.

The injection procedure was used to create composite materials from the melt. Thus, a material based on macromolecular compounds was brought to a flow condition under pressure and inserted into a forming mold, where it was held under pressure and hardened via cooling. The samples were created using a Dr. Boy 35A laboratory micro-extruder from Koenigswinter, Germany. The interface and thermal diagrams of the injection machine are presented in Figure 6, where the sequential temperatures for the PE and PP can be noticed. These samples have the advantage of having reduced internal tensions in the end product due to their injection into an open mold that closes after the injection process is completed.

Three types of samples were created, beginning with ground and regranulated thermoplastic matrices that were derived from electronic waste. To increase their thermal, electrical, and mechanical qualities, the LDPE, PP, and HDPE materials recycled from electronic waste were reinforced with increasing percentages (3%, 7%, and 10%) of WEEE powder, as follows:

- LDPE + 3%-Regranulated LDPE from electronic waste/3% WEEE powder;
- LDPE + 7%-Regranulated LDPE from electronic waste/7% WEEE powder;
- LDPE + 10%-Regranulated LDPE from electronic waste/10% WEEE powder;
- HDPE + 3%-Grinding of HDPE from electronic waste/3% WEEE powder;
- HDPE + 7%-Grinding of HDPE from electronic waste/7% WEEE powder;
- HDPE + 10%-Grinding of HDPE from electronic waste/10% WEEE powder;
- PP + 3%-Regranulated PP from electronic waste/3% WEEE powder;
- PP + 7%-Regranulated PP from electronic waste/7% WEEE powder;
- PP + 10%-Regranulated PP from electronic waste/10% WEEE powder.

The hydrostatic density was calculated using the XS204 Analytical Balance (Mettler-Toledo AG, Greifensee, Switzerland), which has the following specifications: maximum capacity, 220 g; precision, 0.1 mg; linearity, 0.2 mg; internal calibration; density kit for solids and liquids; and an RS 232 interface. The temperature at work was 25.2 °C.

An X-ray diffractometer type D8 Advance was used to perform polycrystalline material analysis and resonant incident technique thin layer analysis, with a software acquisition and interpretation database for PDF-ICDD. The diffractometer’s technical specifications are as follows: X-ray tube with Cu anode; Ni K filter; step of 0.04^o^; measurement time of 2 s/step; measuring range, 2θ =2–60°.

An SEM equipped with a field emission source and a focused ion beam was used. Element chemical analyses were performed using the EDX type dispersive probe mounted on the microscope to provide information on the point composition on the surface of the analyzed material. As a result, in order to obtain the most accurate information on the composition, different areas of the material’s surface were explored, with the software then allowing for the integration of the obtained compositional information and the formation of an image of the material’s degree of homogeneity in particular.

The simultaneous thermal analyzer TG-DSC type STA 449 F3 Jupiter, NETZSCH, Selb Germany, allows for the determination of mass variations and thermal changes for many types of materials, including inhomogeneous materials. The technical specifications are as follows: a temperature range of −150 °C–1550 °C; heating speeds of 0.1–50 °C min; a cooling time of 1500–50 °C <30 min; a balance maximum capacity of 35 g; a balance resolution of 1 µg; a DSC resolution < 1 µW (depending on the sensor); a working atmosphere including inert, oxidizing, reducing, static, and dynamic states; and a vacuum system of a maximum of 10^−2^ mbar.

The thermal conductivity was measured with the LFA 447 Nanoflash device (Netzsch, Selb, Germany). The thermal diffusivity of a material is a thermophysical property that determines the speed of heat propagation via conduction during the variation of temperature with time. The higher a material’s thermal diffusivity is, the faster the heat propagation is. A thermal diffusivity of between 25 °C and 95 °C was determined with an LFA 447 NanoFlash–Netzsch (Germany) device, according to the ASTM E-1461:2007 standard [49], using the “flash” method. A powerful xenon lamp was used as the radiation energy source, and the irradiation time on the front face of the sample was 0.18 ms. The samples were analyzed three times at each temperature. The increase in temperature on the other surface of the sample was measured with the help of an InSb type infrared (IR) detector.

## 3. Results and Discussion

### 3.1. Physical Property Testing and Hydrostatic Density Determination

According to the results in Table 3, adding increasing percentages of nanoconductive powder enhances the density of the composite material. The HDPE composite material milled with 10% WEEE powder has the highest density of all the studied samples, whereas the LDPE regranulated from electronic waste has the lowest density with 3% WEEE powder.

### 3.2. Identifying Crystalline Phases

**Figure 7:**
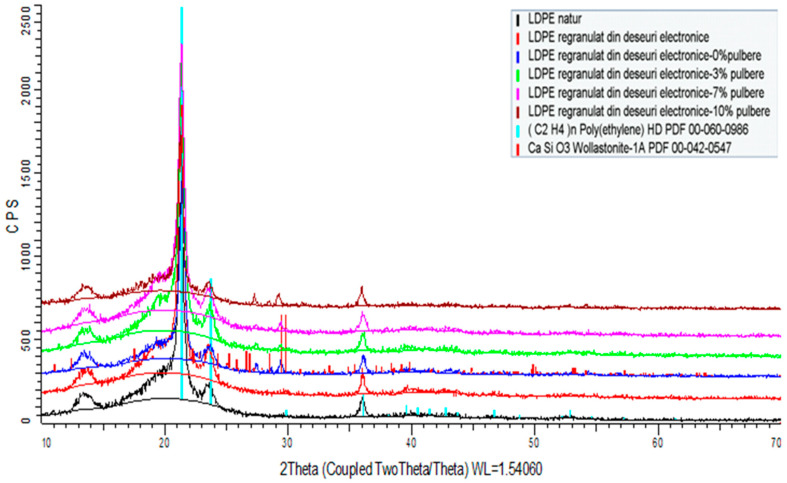
XRD spectra of composites including LDPE as the basis polymer.

**Figure 8:**
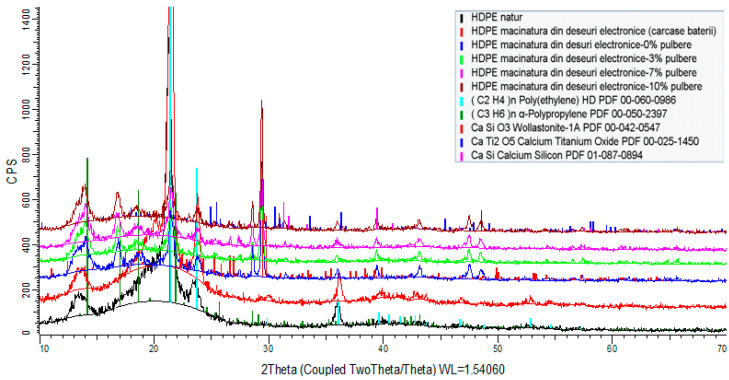
XRD spectra of composites including HDPE as the basis polymer.

**Figure 9:**
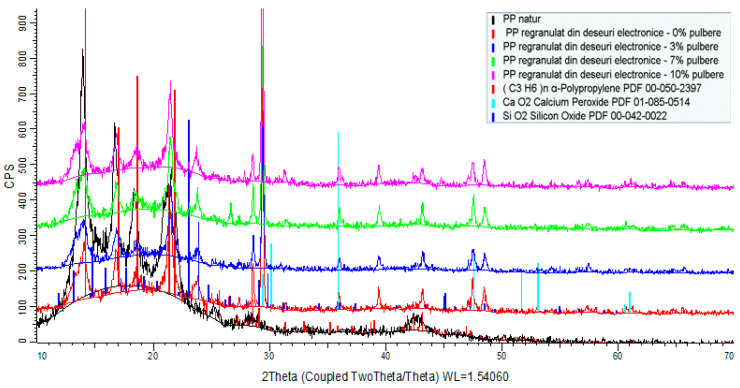
XRD spectra of composites including PP as the basis polymer.

Figure 7, Figure 8 and Figure 9 illustrate the spectra of the composite materials made from the basic polymers LDPE, HDPE, and PP. The powder enhances the density of the composite material. The HDPE composite material milled with 10% WEEE powder has the highest density of all the studied samples, whereas the LDPE regranulated from electronic trash has the lowest density with 3% WEEE powder. The analysis identifies chemicals that have a crystalline state and are present in at least 3% quantities in the material to be tested. As a result, this approach cannot identify chemicals that are amorphous or have very low concentrations.

The X-ray diffraction study of all composite material samples produced under the contract indicated the following:

- High-density polyethylene (re-granulated HDPE from electronic trash) product samples:
  - The basic polymer composition is made up of a blend of high-density polyethylene and residual polypropylene (about 4%);
  - Adding conductive nanopowder resulted in the emergence of peaks specific to the compounds present (calcium oxides, titanium oxides, silicon oxides, and/or their mixtures).
- Samples of regranulated low-density polyethylene products (regranulated LDPE from electronic waste):
  - The basic polymer composition is entirely composed of low-density polyethylene;
  - The addition of conductive nanopowder resulted in the emergence of distinct peaks.
- Polypropylene-based product samples (regranulated from electronic waste):
  - The fundamental polymer composition consists completely of low-density polyethylene;
  - Peaks specific to the chemicals present (calcium oxides, silicon oxides) occurred with the addition of conductive nanoparticles.

### 3.3. Mechanical Characteristics Determination

#### 3.3.1. Tensile Strength Determination

When the nanoconductive powder is introduced within the polymer matrix, the composite material stiffens, indicating an increase in mechanical resistance but also a decrease in elongation (Table 4). The best mechanical resistance is provided by PP from electronic waste with 10% WEEE powder, whereas HDPE and PP from electronic waste with 3% WEEE powder offer the best flow resistance.

#### 3.3.2. Three-Point Bending Strength Measurement

The mechanical strength obtained via tensile testing for the LDPE samples (regranulated LDPE from electronic waste with the addition of 3%, 7%, and 10% WEEE powder) is nearly the same as the mechanical resistance obtained via three-point bending. The same phenomenon can be noticed when the values of the resistance to flow obtained in the tensile tests—specifically, the three-point bending measurement—are compared to the values of the longitudinal modulus of elasticity (Young’s modulus) obtained in the tensile tests, specifically via three-point bending (Table 5). As a result, the mechanical properties stay fairly constant regardless of the percentage of WEEE powder used. However, when the percentage of added conductive powder increases, the mechanical resistance to bending increases across three points. This mechanical behavior is explained by the fact that when the nanoconductive powder is added to the polymer matrix, the composite material stiffens, indicating an increase in mechanical resistance. It is observed that PP from electronic waste with 10% WEEE powder has the highest mechanical resistance to three-point bending, whereas PP from electronic waste with 3% WEEE powder has the highest flow resistance.

#### 3.3.3. Shore Hardness Determination

The Shore hardness evolution graph shows that no changes exist for the samples of regranulated LDPE from electronic waste with 3%, 7%, and 10% WEEE powder; the Shore hardness value is the same regardless of the percentage of nanopowders (Table 6). There is no significant difference in the measured hardness of the composite materials with increasing percentages of powder. At the same time, the addition of increasing percentages of powder (3%, 7%, and 10%) did not result in a substantial increase in hardness.

### 3.4. Chemical Property Testing

#### 3.4.1. Swelling Degree Determination in Water and Solvent

The swelling capacity in water and solvent (toluene) of the examined compounds was determined using the SR EN ISO 175/2011 technique [37]. Thus, 1 g of the composite material was weighed and deposited in plastic ampoules. Two sets of samples were prepared: one to measure the degree of swelling in water and one for determining the degree of swelling in solvent (toluene). The ampoules containing the composite material were filled with double-distilled water and then with the solvent (toluene) and stored at room temperature for 24 h.

The following formula was used to calculate the degree of swelling:

$$\;Q=\frac{X_{2}-X_{1}}{X_{1}}\times 100$$

where

*Q*—degree of swelling;
*X*_2_—the inflated polymer mass;
*X*_1_—dry polymer mass.

With a 95% confidence level, the degree of swelling was calculated as the average of five measurements done on five different samples, removing out-of-range values. According to the results of the experiments, the PP material regranulated from electronic waste with 10% WEEE powder has the highest degree of swelling. The material with the lowest degree of swelling in water at room temperature, namely the HDPE milled from electronic waste with 3% WEEE powder, was chosen among all the composite materials examined (Table 7), where Δm = *X*_2_ – *X*_1_ is the mass variation after immersion in water.

The degree of swelling in the solvent for all samples analyzed based on LDPE (regranulated LDPE from electronic waste with 3%, 7%, and 10% WEEE powder), HDPE (grinding of HDPE from electronic waste with 3%, 7%, and 10% WEEE powder), and PP (regranulated PP from e-waste with 3%, 7%, and 10% WEEE powder), is higher than the swelling limit in water of the same samples (Table 8). The material variant with the lowest degree of swelling in the solvent at room temperature, namely the regranulated PP from electronic waste/10% WEEE powder, can be chosen as the optimal option for use in conditions of exposure to organic solvents among all the composite materials studied.

#### 3.4.2. X-ray Fluorescence Spectrometry (XRF) Elemental Chemical Analysis

**Figure 10:**
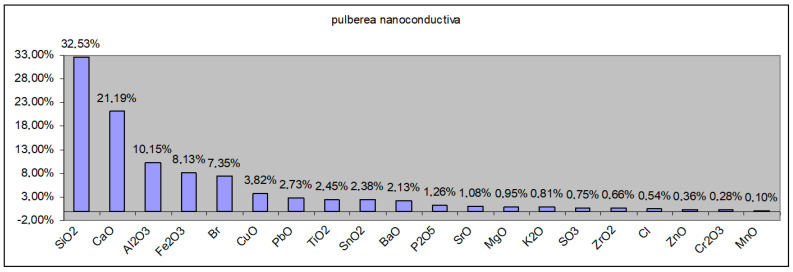
XRF results for WEEE powder component parts.

The results obtained for the samples with the addition of 10% WEEE powder are shown among the samples with the addition of WEEE powder. Chemically, the samples with 3% and 7% additions of WEEE material did not provide any further information. In addition to the polymer matrix, the samples of products based on low-density polyethylene (LDPE range) contain mostly the elements Ca, Ti, Al, Si, Fe, P, Pb (in the form of oxides), Br, and Cl (Figure 10). Aside from the polymer matrix, the samples of products created in the form of grinding based on high-density polyethylene (HDPE range) contain mostly the elements Ca, Ti, Si, Fe, Mg, Pb, P, Cu, Sn (in the form of oxides), Br, and Cl. In addition to the polymer matrix, the samples of the regranulated products based on polypropylene (PP range) comprise mostly the elements Ca, Si, Ti, Fe, Mg, Pb, P, Cu, S, Sn (in the form of oxides), Br, and Cl.

It has also been observed that products based on LDPE regranulated from electronic waste with the addition of WEEE powder contain traces of As, an element that is not present in products based on HDPE or PP; products based on HDPE milled from electronic waste with the addition of WEEE powder as well as products based on regranulated PP from electronic waste with the addition of WEEE powder both contain Mg, and the percentage of Zn in the form of oxide is the same for all products based on LDPE, HDPE, and PP.

It is assumed that the existence of some traces of elements appearing incidentally in some samples is related to the previous processing of the polymer beads.

According to the graph below, the components of the conductive powder are present in considerable percentages in the composition.

To better highlight the elements contained in the examined samples, a graphic representation was created that excluded C (organic) and calcium oxide CaO (as an addition). All of the samples studied include a higher percentage of TiO_2_; the presence of SiO_2_ is also reported. MgO, Fe_2_O_3_, ZnO, Al_2_O_3_, PbO, and CuO are also present in trace amounts.

#### 3.4.3. Chemical Element Analysis—SEM with the EDX Dispersive Probe

This type of examination was carried out using an SEM equipped with a field emission source and a focused ion beam. Element chemical analyses could be performed using the EDX type dispersive probe mounted on the microscope to provide information on the point composition on the surface of the analyzed material. As a result, in order to obtain the most accurate information on the composition, different areas of the material’s surface were explored, with the software then allowing for the integration of the obtained compositional information and the formation of an image of the material’s degree of homogeneity in particular.

It was discovered that the WEEE powder produced via WEEE processing has a significant organic component. Thus, a 10% WEEE powder addition in the composite mass represents a maximum contribution of 6% mineral components, with the rest being organic. The explanation is straightforward and stems from the fact that the powder is derived from the processing of printed circuits, which contain thermosetting elements with a significant content of organic components, as well as conductive and semi-conductive mineral and micro-metallic components.

**Figure 11:**
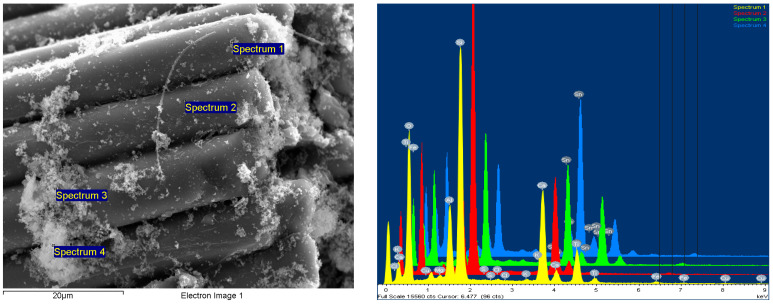
LDPE from e-waste with 10% NC powder composite sample—mag 5000× secondary electron (SE) image with spectra points on the sample, four areas analyzed.

**Figure 12:**
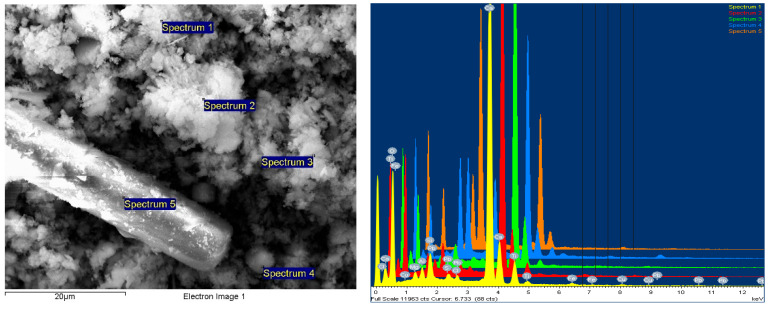
HDPE from e-waste with 10% NC powder composite sample—mag 5000× secondary electron (SE) image with spectra points on the sample, five areas analyzed.

**Figure 13:**
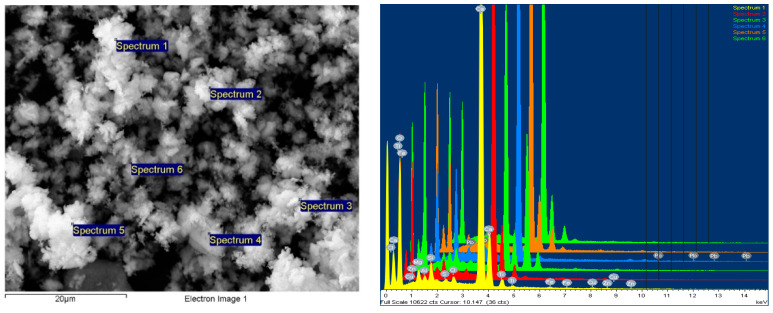
PP from electronic waste with 10% NC powder composite sample—mag 5000× secondary electron (SE) image with spectra points on the sample, six areas analyzed.

It was found that the addition of progressive concentrations of WEEE powder from 3% to 7% and finally 10% determines a corresponding increase in the concentrations of inorganic elements/metal oxides identified in the analysis (Figure 11, Figure 12 and Figure 13 and Table 9).

Finally, the presence of Br in the composition (approx. 0.05%) attests to the fact that brominated flame retardant compounds present in electronic equipment have been successfully stabilized and integrated, which is one of the EC’s requirements for material recycling technologies—to stabilize hazardous materials, which cannot be recycled any other way. Additionally, the flame retardant features of WEEE are partially transferred to the composite materials, which is a clear benefit.

The studied images in Figure 11, Figure 12 and Figure 13 and the compositions—obtained by scanning the composition on all micro-areas of interest—indicate a good homogeneity of the composite structure, attesting to the correct method of dispersing the additives and the thermoplastic processing. It is obvious that the LDPE matrix appears more filamented compared to that of HDPE and PP, which appear more dispersed with lumpy aspects.

However, the ability of such powders to disperse more uniformly, avoid reagglomeration/sedimentation, and have an increased affinity towards the polyolefin thermoplastic matrix makes them more appealing for use in customized composites for automotive applications. The presence of residual organic components from thermoset resins dispersed and linked among the inorganic components is beneficial, and ease the uniform dispersion within the matrix, with the phenomenon being similar to that of pre-composition—an eventual coating at the nano/micro scale of mineral components with organic particles, which is, in fact, a classical procedure to ease the uniform dispersion of additives within a thermoplastic matrix.

### 3.5. Thermal Property Analysis

The mathematical analysis of the distribution of the temperature variation as a function of time allows the determination of the thermal diffusivity “α”. This is done via the analysis software of the device that allows for the manual or automatic control of the experimental process, as well as the evaluation of the results. The software contains several mathematical models for this application. The simplest model is the “adiabatic model”. For this model, the thermal diffusivity is calculated according to the relationship below [2]:

$$\;\alpha =0.1388\times \frac{I^{2}}{t_{1/2}}$$

where

*α* = thermal diffusivity (mm^2^/s);
*I* = sample height (mm);
*t*_1/2_ = the time (s) when the temperature rises to half, measured on the other side of the sample.

The “flash” method is advantageous due to the simple geometry and small size requirements of the samples, the speed of the measurements, and the ease of use. For accurate measurements, it is recommended that the samples be cylindrical or parallelepiped, and have flat surfaces and parallel faces [2].

Specific heat (also called mass heat capacity) represents the amount of heat required per unit of mass (kg) of a homogeneous body to change its temperature by one degree, and is expressed according to the relationship below [2]:

$$\;C_{P}=\frac{Q}{m\times ∆T}$$

where

*C_P_* = specific heat (J/kg·K);
*Q* = heat (J);
*m* = sample mass (kg);
Δ*T* = temperature variation of the sample (K).

This technique involved a comparison between the temperature increase of the sample due to the pulse emitted by the xenon lamp (the voltage of the final detector minus the voltage of the baseline detector), and the temperature increase of the standard sample, tested at the same time and under the same conditions. In this way, the specific heat and the thermal diffusivity of the sample could be measured after a single analysis.

Thus, the specific heat of the sample was determined according to the following equation [2]:

$$C_{P}=\frac{{\left(mC_{P}∆T\right)}_{standard}}{{\left(m∆T\right)}_{sample}}=\frac{{\left(mC_{P}∆V\right)}_{standard}G_{standard}}{{\left(m∆V\right)}_{sample}\times G_{sample}}$$

where

*V* = voltage variation recorded by the detector (proportional to T) (V);
*G* = detector gain, which is a constant equal to 50,020.

#### Thermal Conductivity Measurement

Thermal conductivity is the physical quality that characterizes the ability of a material to transmit heat when it is subjected to a temperature difference.

Thermal conductivity is defined for a mass body that has a temperature gradient, and represents the heat flow that crosses a unit of transversal surface, in a unit of time, in the direction of the unit temperature gradient. The thermal conductivity of the samples was determined with the following equation [2]:

$$\lambda \;=\;\alpha \;\times \;C_{P}\;\times \;d$$

where

λ = thermal conductivity (W/m·K);
*α* = thermal diffusivity (m^2^/s);
*C_P_*= specific heat (J/kg·K);
*d* = density (kg/m^3^).

From the values from Table 10 and Table 11, we can draw the conclusion that by adding WEEE powders, the highest thermal conductivity value is obtained for materials with an addition of 10% WEEE powder. It can be observed that the highest thermal conductivity value is recorded for regranulated PP from electronic waste with 10% addition of WEEE powder.

From the analysis of the thermogravimetric data, presented in Table 12 it was observed for the composites made from recycled matrices with powder—compared to the matrices of origin—that the composite materials show a tendency to translate the thermal phenomena towards higher temperatures. This occurrence is confirmed by the temperature differences identified in the glass transition process, where the increase in the temperature at the beginning of the process can be observed (e.g., LDPE—initial T = 203.9 °C, compared to the composite based on LDPE with initial T = 230.4 °C), which is due to the addition of the conductive powder.

These temperature values will constitute the starting basis for the technology scaling process by operating the screw extruder in a double adiabatic process in order to avoid scaling problems due to differences in heat transfer.

### 3.6. Dielectric Properties

A Novocontrol measuring device was used for the dielectric measurements [50], and includes the following:

- Novocontrol AlphaN, broadband dielectric analysis stand;
- Novocontrol BDS 1200, calibrated cells (max. freq. 8 GHz);
- QUATRO-Cryosystem: cooling and heating system with liquid nitrogen (−160 °C ÷ +400 °C);
- WinDETA/WinFIT—software package for measurement, calibration, and analysis;
- Rhode–Schwartz NVR Network Analyzer, frequency range 20 kHz ÷ 8 GHz, impedance 0.1 Ω ... 10 kΩ, tan(δ) accuracy > 3 × 10^−2^.

**Figure 14:**
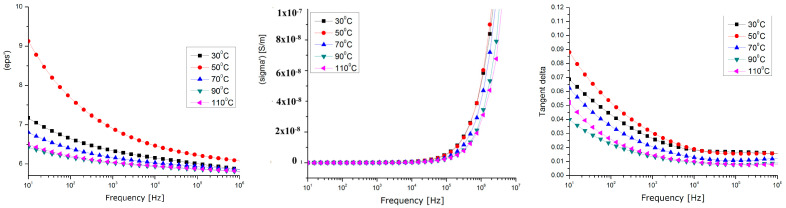
Dielectric measurements for HDPE composites with 10% powder content.

**Figure 15:**
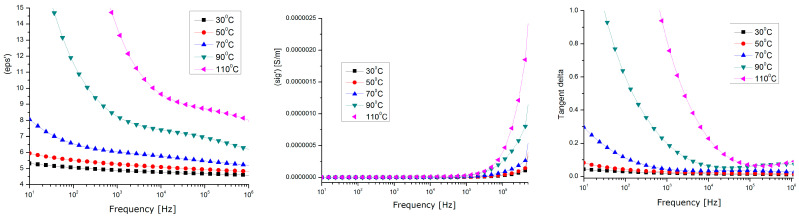
Dielectric tmeasurements for LDPE composites with 10% powder content.

**Figure 16:**
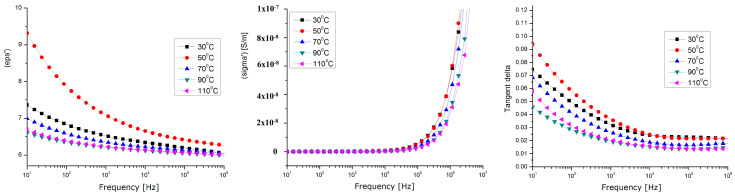
Dielectric measurements for PP composites with 10% powder content.

Dielectric tests (Figure 14, Figure 15 and Figure 16) were performed only for the composites with a 10% powder content due to their relevant behavior.

The analyzed dielectric properties were the relative permittivity (eps’), real conductivity (sigma’), and dielectric loss expressed as Tgδ (Tangent Delta).

By correlating the dielectric characteristics obtained for the three samples of composites with their physico-chemical properties, it can be found that the values of the dielectric characteristics vs. temperature corresponding to the samples of HDPE and PP are relatively similar (Figure 14, Figure 15 and Figure 16), but different values were achieved at higher temperatures for the sample of LDPE (the tg. Delta and the real permittivity were higher at over 70 °C). This may be explained by the fact that, in lower-density polyethene which has a lower viscosity at higher temperatures, the metallic components of the powder, i.e., Zn, Sn, and Pb, become more mobile and partially separate themselves from the oxide components, and so they act as higher-energy polarization centers, or higher-energy loss centers, due to a higher interfacial polarization effect at lower frequencies (with tg. Delta values of about one, even at the kHz frequency domain for LDPE).

In all cases, the maximum polarization effect is achieved at a temperature of approx. 50 °C, which may be explained by the optimal polarization at the interface between the polymer and the powder, when the thermal movement achieves its optimum level. In this context, considering the perspectives of the current research, the very high share of WEEE of electronic equipment produced after the 2000s (with a high degree of integration, in which there is a significantly large number of IC-type active components, and the passive ones are of a volume proportion below 30% compared to the active ones), and the future trends of electronic technology, the methods for the integral recycling of WEEE towards components with higher value in the context of a circular economy must become a stringent preoccupation of scientists.

The composite materials presented above, due to their relevant dielectric properties, may be further tested for applications in electromagnetic shielding at frequencies above 1 kHz, or for electromagnetic interference/electromagnetic compatibility (EMI/EMC and ESD) applications at lower frequencies, due to their superior dielectric loss factor values, which are associated with relevant behavior around exploitation temperatures, mainly for the electric, electronic, or automotive industries.

The obtained dielectric performance is in line and comparable with the results for similar composites from previous research, as presented in [2,31,32,33,34,35,36,37], but in our case, the main advantages are related, one side, to the integral recycling of WEEE towards an innovative product under the concept of a circular economy, and on the other side, to a clear economic benefit by substituting the very expensive powders that are actually used for applications in electromagnetic shielding with very cheap homologue powders obtained from the non-metallic thermoset components of WEEE (electronic components).

Hence, the presence of mineral additions is advantageous for improving mechanical, thermal, and electrical features, as well as, from the perspective of creating specific composite materials for automotive purposes, raising the fire resistance of the corresponding products.

## 4. Conclusions

Considering the concept of a circular economy and the perspectives of the current research, the very high share of waste electronic equipment produced after the 2000s (with a high degree of integration, in which there is a significantly large number of IC-type active components, and the passive ones are of a volume proportion below 30% compared to the active ones), and the future trends of electronic technology, the methods for the integral recycling of WEEE become an imperative purpose. Our paper suggests a method for the integrated recycling of thermoplastic and non-metallic components of WEEE, after all classical recycling processes are accomplished.

A relevant stage was dedicated to obtaining powder from the thermoset components of WEEE (electronic components), which was characterized by physical–chemical procedures and provided to include relevant components to be further used as additives in thermoplastic composites. The second stage referred to manufacturing and characterizing composites made of recycled LDPE, HDPE, and PP from WEEE, with up to a 10% addition of non-metallic powder. The composites proved a homogenous structure, with high chemical and thermal stability.

Finally, the dielectric properties were tested, and high values for the dielectric permittivity and loss factor (tgδ) were achieved. For all matrices, the maximum polarization effect was achieved at a temperature of approx. 50 °C, which may be explained by the optimal polarization at the interface between the polymer and the powder, when the thermal movement achieves its optimum level. The composite materials with up to a 10% addition of non-metallic powder, due to their relevant dielectric properties, may be further tested as good candidates for applications in electromagnetic shielding at frequencies above 1 kHz, or for electromagnetic interference/electromagnetic compatibility (EMI/EMC and ESD) applications at lower frequencies, due to their superior dielectric loss factor values, associated with the relevant behaviors around exploitation temperatures, mainly for the electric, electronic, or automotive industries.

The main advantages of this study are related, on one side, to the integral recycling of WEEE towards an innovative product under the concept of a circular economy, and on the other side, to a clear economic benefit by substituting the very expensive powders that are actually used for applications in electromagnetic shielding with very cheap homologue powders obtained from the non-metallic thermoset components of WEEE (electronic components). On the other hand, it was noticed that the brominated flame retardant compounds present in electronic equipment were successfully stabilized and integrated within composites, which is one of the EC’s requirements for material recycling technologies—to stabilize hazardous materials, which cannot be recycled any other way.

## Figures and Tables

**Table 1:** The freely poured density of powder (ρ) as a function of powder weight.

Nr. Test	Hole Size (mm)	Powder Weight (mg)	ρ_a_ = m/V_a_ (g/cm^3^)	ρ_average_ (g/cm^3^)
1	5	3.0234	0.6694	0.6630
2		3.0321	0.6713	
3		2.9886	0.6617	
4		2.9346	0.6497	

**Table 2:** Powder components.

Formula	Z	Concentration (%)	The Most Intense Peak	The Net Intensity of the Signal	Statistical Error (%)	Lower Limit of Detection	The Thickness of the Analyzed Layer
CaO	20	25.16	Ca KA1-HR-Tr	25.2	0.625	282.3 PPM	42 μm
Fe_2_O_3_	26	15.72	Fe KA1-HR-Tr	15.72	0.409	126.0 PPM	101 μm
CuO	29	13.91	Cu KA1-HR-Tr	13.9	0.657	126.4 PPM	145 μm
SiO_2_	14	12.69	Si KA1-HR-Tr	12.7	1.61	478.4 PPM	7.2 μm
SnO_2_	50	7.93	Sn KA1-HR-Tr	7.93	0.865	699.6 PPM	1.80 mm
PbO	82	7.68	Pb LB1-HR-Tr	7.683	0.528	219.1 PPM	0.37 mm
BaO	56	3.39	Ba LA1-HR-Tr	3.39	2.73	574.9 PPM	44 μm
Br	35	3.10	Br KA1-HR-Tr	3.105	0.504	78.5 PPM	0.31 mm
Cr_2_O_3_	24	2.68	Cr KA1-HR-Tr	2.68	1.35	133.7 PPM	69 μm
ZnO	30	2.61	Zn KA1-HR-Tr	2.61	0.652	73.2 PPM	172 μm
MnO	25	1.88	Mn KA1-HR-Tr	1.88	1.27	133.0 PPM	85 μm
NiO	28	1.57	Ni KA1-HR-Tr	1.57	2.06	97.2 PPM	119 μm
ZrO_2_	40	0.69	Zr KA1-HR-Tr	0.532	1.22	134.7 PPM	0.52 mm
SrO	38	0.61	Sr KA1-HR-Tr	0.605	1.24	70.7 PPM	0.42 mm
Ag	47	0.38	Ag KA1-HR-Tr	0.38	8.77	381.5 PPM	1.25 mm

**Table 3:** Results of hydrostatic density determination.

Material	Density (g/cm^3^)
LDPE	0.893
LDPE + 3%	0.902
LDPE + 7%	0.903
LDPE + 10%	0.952
HDPE	0.929
HDPE + 3%	0.935
HDPE + 7%	0.941
HDPE + 10%	1.02
PP	1.001
PP + 3%	1.003
PP + 7%	1.005
PP + 10%	1.006

**Table 4:** Tensile strength for the tested materials.

Material	Mechanical Resistance (MPa)	Flow Resistance (MPa)	Elongation A (%)	Young’s Modulus (GPa)
LDPE	12.9	2.13	351.1	0.16
LDPE + 3%	13.2	2.06	349.5	0.14
LDPE + 7%	13.54	1.81	342.26	0.1
LDPE + 10%	14.03	0.66	339.87	0.07
HDPE	14.23	8.82	0.29	0.61
HDPE + 3%	14.61	8.68	0.26	0.59
HDPE + 7%	15.77	7.37	0.24	0.57
HDPE + 10%	15.93	6.34	0.17	0.56
PP	17.89	8.53	0.28	0.69
PP + 3%	18.03	8.42	0.21	0.65
PP + 7%	18.89	8.17	0.18	0.64
PP + 10%	19.66	8.08	0.17	0.57

**Table 5:** The three-point bending strength of all materials.

Material	Mechanical Resistance (MPa)	Flow Resistance (MPa)	Young’s Modulus (GPa)
LDPE	16.34	2.32	0.17
LDPE + 3%	16.85	2.79	0.2
LDPE + 7%	17.48	3.1	0.29
LDPE + 10%	18.23	3.25	0.33
HDPE	38.02	22.76	1.00
HDPE + 3%	38.67	24.56	1.01
HDPE + 7%	38.82	20.17	1.41
HDPE + 10%	39.07	20.07	1.45
PP	35.09	26.83	1.23
PP + 3%	36.49	27	1.67
PP + 7%	38.07	24.07	1.36
PP + 10%	45.18	21.02	1.53

**Table 6:** The results of the Shore hardness determination.

Material	Shore Hardness A (HS)
LDPE	97
LDPE + 3%	97
LDPE + 7%	97
LDPE + 10%	97
HDPE	97
HDPE + 3%	97
HDPE + 7%	98
HDPE + 10%	98
PP	95
PP + 3%	95
PP + 7%	95
PP + 10%	97

**Table 7:** The results of the swelling tests in water at room temperature.

Material	*X*_1_	*X*_2_	∆m	*Q*
LDPE	1.2156	1.4453	0.0179	2.012
LDPE + 3%	1.4259	1.4563	0.0213	2.132
LDPE + 7%	1.4351	1.4919	0.0396	3.9579
LDPE + 10%	1.4512	1.5004	0.0339	3.3903
HDPE	1.3903	1.3981	0.0083	0.8103
HDPE + 3%	1.3927	1.4051	0.0089	0.8904
HDPE + 7%	1.3975	1.425	0.0197	1.9678
HDPE + 10%	1.4035	1.4237	0.0144	1.4393
PP	1.3698	1.3902	0.0187	2.0951
PP + 3%	1.3739	1.4064	0.0237	2.3655
PP + 7%	1.3711	1.3927	0.0158	1.5754
PP + 10%	1.3946	1.4979	0.0741	7.4071

**Table 8:** The results of the swelling tests in solvent (toluene) at room temperature.

	LDPE + 10%	HDPE + 10%	PP + 10%
C (organic)	93.80%	92.50%	90.30%
CaO	3.75%	5.41%	7.18%
TiO_2_	0.63%	0.53%	0.49%
SiO_2_	0.58%	0.51%	0.49%
Al_2_O_3_	0.47%	0.10%	0.10%
Cl	0.11%	0.16%	0.16%
Fe_2_O_3_	0.19%	0.19%	0.16%
MgO	0.10%	0.11%	0.14%
PbO	0.14%	0.10%	0.10%
P_2_O_5_	0.07%	0.09%	0.08%
Br	0.03%	0.09%	0.10%
CuO	0.10%	0.08%	0.07%
SnO_2_	0.04%	0.05%	0.05%
ZnO	0.02%	0.02%	0.02%
SrO	0.0046%	0.01%	0.02%
ZrO_2_	0.0026%	0.01%	0.01%
SO_3_	0.00%	0.00%	0.06%
Cr_2_O_3_	0.00%	0.01%	0.00%
As_2_O_3_	0.0036%	0.00%	0.00%

**Table 9:** EDAX results for all samples.

	O	Mg	Al	Si	S	Cl	K	Ca	Ti	Fe	Cu	Zn	Pb
LDPE + 3%	49.19	0.95	20.26	42.78	0.77	1.13	4.35	57.63	32.03	0.48	0.48	0.75	1.56
LDPE + 7%	46.29	13.07	0.83	23.68	0.36	0.55	62.9	6.83	1.53	0.61	4.38	1.93	46.29
LDPE + 10%	54.89	0.94	7.98	25.82	0.22	0.39	0.32	18.81	15.04	0.78	0.22	8.74	54.89
HDPE + 3%	58.76	1.34	8.17	27.04	0.19	1.09	50.65	2.56	0.5	0.47	0.82	58.76	1.34
HDPE + 7%	57.83	1.91	0.29	1.31	0.39	0.57	57.7	2.51	0.31	0.37	57.83	-	-
HDPE + 10%	53.26	1.76	9.16	26.05	0.27	9.33	48.73	3.87	0.7	2.11	1.17	53.26	1.76
PP + 3%	57.63	8.16	0.67	1.08	0.26	0.71	44.59	16.56	0.23	0.63	0.9	1.74	57.63
PP + 7%	57.83	1.91	0.29	1.66	0.39	0.57	56.7	10.56	2.13	0.37	57.83	-	-
PP + 10%	55.54	12.77	7.8	25.81	0.32	0.65	47.57	4.66	1.24	0.54	0.96	55.54	12.77

**Table 10:** The experimental results regarding glass transitions for all analyzed materials.

Material	Melting	Glass Transitions		
	Q (J/g)	Cp J/g·K	Initial Temperature (°C)	Final Temperature (°C)
LDPE	92.86	0.102	230.9	249.0
LDPE + 3%	93.82	0.121	230.4	248.9
LDPE + 7%	196.5	0.462	224.3	239.6
LDPE + 10%	90.43	-	-	-
HDPE	46.02	1.932	221	230
HDPE + 3%	46.86	1.951	225	234
HDPE + 7%	48.32	-	-	-
HDPE + 10%	59.61	1.111	229.1	238.7
PP	62.00	0.800	247.3	253.8
PP + 3%	62.45	0.718	246.9	254.9
PP + 7%	54.86	2.962	232	239.6
PP + 10%	62.57	0.407	249.8	259.3

**Table 11:** The results of the thermal conductivity determination.

Material	Diffusivity (mm^2^/s)	Conductivity (W/(m·K))	Cp (J/g/K)
LDPE + 3%	0.197	0.179	2.551
LDPE + 7%	0.22	0.202	2.327
LDPE + 10%	0.226	0.208	2.275
HDPE + 3%	0.198	0.18	1.716
HDPE + 7%	0.2	0.182	2.155
HDPE + 10%	0.24	0.222	1.733
PP + 3%	0.223	0.205	1.597
PP + 7%	0.231	0.213	1.766
PP + 10%	0.273	0.255	2.018

**Table 12:** The results of the analysis of the thermogravimetric data.

Material Matrix + x% Powder	Air/Static Conditions										
	Process I Melting	Process II Oxidation			Process III Thermo-Oxidatuion			Process IV Decomposition			%Δm Total
	T_min_ DSC, °C	T_max_ DSC, °C	T_DTG_, °C	%Δm	T_max_ DSC, °C	T_DTG_, °C	%Δm	T_max_ DSC, °C	T_DTG_, °C	%Δm	
LDPE	116	413	474	97	517	-	-	662	664	-	97
	122										
LDPE + 3%	118	415	474	97	516	-	-	665	665	-	98
	123										
LDPE + 7%	115	418	474	97	514	-	-	681	680	-	99
	124				530						
LDPE + 10%	116	406	474	87	521	-	-	672	672	-	89
	126										
HDPE	130	397	430	81	486	486	3.65	701	701	8.07	89
	165										
HDPE + 3%	131	399	432	81	489	489	3.69	702	702	8.08	89
	166	429	457								
HDPE + 7%	130	385	431	82	490	488	3.64	700	701	8	88
	165	423									
HDPE + 10%	130	379	427	75.24	473	492	3.71	696	700	5.32	84.43
	165	422			488						
PP	130	401	455	80.02	-	-	-	708	708	6.09	88.71
	163	425									
PP + 3%	130	402	456	79.52	-	-	-	709	708	6.12	88.93
	165	426									
PP + 7%	129	297	385	72.12	482	482	6.32	707	702	6.32	84.76
	164	398	398								
		420	427								
PP + 10%	129	438	457	-	487	-	-	708	708	6.57	84.41
	163				514						
